# Decrease in IL‐10 and increase in TNF‐*α* levels in renal tissues during systemic inhibition of nitric oxide in anesthetized mice

**DOI:** 10.1002/phy2.228

**Published:** 2014-02-10

**Authors:** Purnima Singh, Alexander Castillo, Dewan S. A. Majid

**Affiliations:** 1 Department of Physiology, Hypertension & Renal Center of Excellence, Tulane University Health Sciences Center, New Orleans, 70112, Louisiana

**Keywords:** IL‐10, kidney, l‐NAME, nitric oxide synthase, SNAP, TNF‐*α*

## Abstract

Earlier, we demonstrated that the inhibition of nitric oxide synthase (NOS) by nitro‐l‐arginine methyl ester (l‐NAME) infusion increases the endogenous production of proinflammatory cytokine, tumor necrosis factor (TNF‐*α*). In the present study, we examined the hypothesis that inhibition of nitric oxide (NO) production leads to the suppression of interleukin (IL)‐10 (anti‐inflammatory cytokine) generation which facilitates the enhancement of TNF‐*α* production endogenously. Using appropriate enzyme‐linked immunosorbent assay kits and immunohistochemical staining, the levels of IL‐10 and TNF‐*α* in plasma (P) and in renal tissues (R) were measured in anesthetized mice (C57BL/6; ~10 weeks age; *n* = 6/group) infused with or without l‐NAME (200 *μ*g/min/kg; i.v. for 2 h). Compared to vehicle‐treated control mice, l‐NAME‐treated mice had a lower level of IL‐10 (P, 0.3 ± 0.1 vs. 2.6 ± 0.6 ng/mL; R, 0.5 ± 0.1 vs. 3 ± 0.1 ng/mg protein) and a higher level of TNF‐*α* (P, 432 ± 82 vs. undetected pg/mL; R, 58 ± 7 vs. 6 ± 5 pg/mg protein). IL‐10 protein expression, present mostly in the distal nephron segments in control mice, was markedly downregulated in l‐NAME‐treated mice. Compared to control mice, TNF‐*α* expression increased 2.5‐fold in renal cortical sections (mostly in the distal nephron segments) in l‐NAME‐treated mice. Coinfusion of a NO donor, *S*‐nitroso‐*N*‐acetyl‐penicillamine (SNAP; 25 *μ*g/min/kg) with l‐NAME in a separate group of mice prevented these changes in IL‐10 and TNF‐*α* induced by l‐NAME. IL‐10 infusion (0.075 ng/min/g) in l‐NAME‐treated mice markedly attenuated l‐NAME‐induced increments in TNF‐*α*. Thus, these results demonstrate that NOS inhibition decreases endogenous IL‐10 generation and thus, minimizes its immune downregulating action on the TNF‐*α* production in the kidney.

## Abstract (precis)

In this study in mice, we examined the hypothesis that NOS inhibition reduces the endogenous production of anti‐inflammatory cytokine, IL‐10 which facilitates enhanced production of TNF‐α in the kidney. Using appropriate enzyme‐linked immunosorbent assay kits and immunohistochemical staining, levels of IL‐10 and TNF‐α in renal tissue were measured in anesthetized mice infused with or without l‐NAME (200 μg/min/kg) in the presence or absence of a NO donor, *S*‐nitroso‐*N*‐acetyl‐penicillamine (SNAP; 25 μg/min/kg) or IL‐10 infusion (0.075 ng/min/g). These results demonstrate that NOS inhibition decreases endogenous IL‐10 generation and thus, minimizes its immune downregulating action on the TNF‐α production in the kidney.

## Introduction

Nitric oxide (NO), though originally identified as an endogenous vasodilator that acts as a key modulator of cardiovascular functions, is also considered a potent anti‐inflammatory agent. Many recent studies have suggested a direct relationship between NO deficiency and the generation of inflammatory cytokines including tumor necrosis factor‐*α* (TNF‐*α,* Livonesi et al. 2009; Bougaki et al. 2010). Consistent with these findings, our laboratory also reported that systemic infusion of a NO synthase (NOS) inhibitor, nitro‐l‐arginine‐methyl ester (l‐NAME) in anesthetized mice increased plasma level of TNF‐*α* (a proinflammatory cytokine) and increased renal tissue expression of TNF‐*α* protein (Yang et al. 2008). It has been shown earlier that NO downregulates proinflammatory protein and mRNA expression during acute lung injury by an effect upstream of the transcription factor nuclear factor kappa B (NF‐kB), which binds to the promoter region of the proinflammatory cytokine genes (Walley et al. 1999). NO also induces inhibition of NF‐kB in human vascular endothelial cells (Peng et al. 1995). However, this immunomodulatory role of NO is independent of guanylate cyclase (Peng et al. 1995; Walley et al. 1999). This suggests that NO‐induced decreases in proinflammatory cytokines involves additional regulatory pathways independent of guanylate cyclase. However, the mechanism of how NO suppresses the generation of TNF‐*α* or other proinflammatory cytokines remains to be examined further.

In inflammatory conditions in the kidney, cytokines such as TNF‐*α* and interleukin (IL)‐1 are produced by both resident cells (including renal tubular cells) and infiltrating monocytes/macrophages (Sedor 1992; Sedor et al. 1993). TNF‐*α* reduces renal blood flow (RBF) and glomerular filtration rate (GFR) by acting as a vasoconstrictor and can cause natriuresis by inhibiting renal epithelial sodium channel (ENaC) activity (Shahid et al. 2008, 2010; Majid 2011). TNF‐*α* participates in the process of renal injury by recruiting and activating inflammatory cells (Egido et al. 1993). However, besides the production of proinflammatory cytokines, it is possible for an anti‐inflammatory response to contain the inflammation and limit cellular destruction. A key cytokine that appears to restore the balance between proinflammatory and anti‐inflammatory cytokines is IL‐10 (Wang et al. 1995), which is a potent immunoregulatory cytokine produced by macrophages, T cells, B cells, epithelial cells, and mast cells. IL‐10 has been shown to exert a protective effect against inflammation (Bean et al. 1993; Gerard et al. 1993) due to generalized downregulation of proinflammatory cytokines such as IL‐1, TNF‐*α*, and IL‐6 (Florentino et al. 1991). However, the regulation of IL‐10 during alteration of NOS activity is not clearly described.

In this study, we examined the hypothesis that inhibition of NO production leads to the suppression of IL‐10 generation which facilitates the enhancement of TNF‐*α* production endogenously. Accordingly, we measured and correlated the changes in the plasma and renal tissue levels of IL‐10 and TNF‐*α* during systemic inhibition of NO production in mice. Samples of plasma and renal tissue were collected from anesthetized mice treated with or without systemic administration of NOS inhibitor, l‐NAME. To determine whether or not an inhibition of NO production is directly involved in l‐NAME‐induced changes in the production of these cytokines, their levels were also assessed during replacement of NO by the administration of S‐nitroso‐N‐acetylpenicillamine (SNAP; a NO donor) in l‐NAME pretreated mice. To evaluate a direct contributory role of IL‐10 reduction in the enhancement of TNF‐*α* production during NOS inhibition, plasma and renal tissue levels of TNF‐*α* were also assessed during replacement of IL‐10 (mouse recombinant IL‐10) by administering it exogenously in l‐NAME pretreated mice.

## Methods

### Animal preparations

All the experimental procedures described in this study were performed in accordance with the guidelines and practices established by the Tulane University Animal Care and Use Committee. C57BL/6 strain male mice (stock no # 000664; Jackson Laboratories, Bar Harbor, ME), aged between 10 and 12 weeks, were housed in a temperature‐ and light‐controlled room and allowed free access to a standard diet (Ralston Purina, St. Louis, MO) and tap water.

On the day of experiments, the mice were anesthetized with inactin (150 mg/kg, i.p.). The plasma and renal tissue samples were collected from these anesthetized mice at the end of the experimental periods with intravenous infusion of different drugs (l‐NAME, 200 *μ*g/min/kg; SNAP, 25 *μ*g/min/kg; IL‐10, 0.075 ng/min/g). The mice were also surgically prepared for renal clearance studies to assess the changes in renal hemodynamic and excretory function due to acute systemic administration of these drugs as described previously (Shahid et al. 2010).

Mice were divided into four experimental groups (*n* = 6 in each group) as follows:

Group 1: Control group – Vehicle (saline) treatment only
Group 2: l‐NAME‐treated group
Group 3: l‐NAME + SNAP‐treated group (SNAP was infused to examine whether or not the l‐NAME‐induced responses on the cytokine levels were reversed by NO replacement)
Group 4: l‐NAME + IL‐10‐treated group (As it was observed in Group 2 that l‐NAME decreased IL‐10 level and increased TNF‐*α*, exogenous IL‐10 was infused to see whether or not the l‐NAME‐induced responses on TNF‐*α* were reversed by IL‐10 replacement)

**Figure 1.**
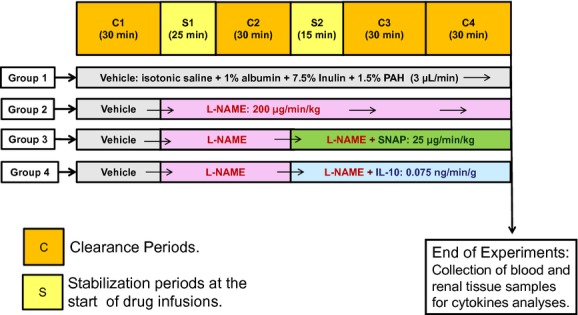
Schematic diagram of the experimental protocol followed in the present study. C1‐4, Clearance periods for 30 min duration each. S1‐2, Stabilization periods preceding each drug infusion. l‐NAME, nitro‐l‐arginine methyl ester; SNAP, S‐nitroso‐N‐acetylpenicillamine; IL‐10, interleukin‐10.

The detailed protocol for each group is described later in “Experimental Protocol” and has been illustrated in Figure 1.

### Experimental protocol

In Group 1 mice, saline as vehicle (with 1% albumin, 7.5% inulin, and 1.5% paraaminohippuric acid [PAH]) was infused throughout the experimental period and urine collections were made for four clearance periods (C1, C2, C3, and C4) of 30 min each as illustrated in Figure 1. A blood sample of 50–70 *μ*L was collected at the end of C1, C2, and C4 for the measurement of inulin and PAH clearance to determine GFR and RBF measurements. In Group 2 mice, l‐NAME was infused (C2, C3, and C4) after the first clearance (C1) period with vehicle infusion. In Group 3 mice, SNAP was infused along with l‐NAME during the last two clearance periods (C3 and C4) after the first two clearance periods with only saline (C1) and only l‐NAME infusion (C2), respectively. Similarly, in Group 4 mice, IL‐10 was infused along with l‐NAME during the last two clearance periods (C3 and C4) after the first two clearance periods with only saline (C1) and only l‐NAME infusion (C2), respectively.

For the measurement of plasma cytokine levels in these experimental animals, a larger sample of blood (about 1 mL) was collected from the carotid cannula after completion of the experimental protocol. This collected blood was immediately centrifuged to separate the plasma and stored at −80°C until analyzed. At the end of the experiment, the animals were euthanized; the kidneys were removed, and weighed. One kidney was stored at −80°C for renal cytokine measurement and the other kidney was fixed in 10% formalin solution and paraffin‐embedded for immunohistochemical (IHC) studies. For measuring renal cytokine levels, a chunk of kidney tissue (about 100 mg in weight) was homogenized in sterile phosphate‐buffered saline containing protease inhibitor at 4°C. Kidney homogenates were centrifuged at 9000*g* for 10 min at 4°C. Supernatants were transferred to clean microcentrifuge tubes and stored at −80°C until analyzed.

### Analysis of samples

#### Enzyme‐linked immunosorbent assay for IL‐10 and TNF‐α level

Levels of IL‐10 and TNF‐*α* in plasma and supernatants from kidney tissue homogenates were measured by enzyme‐linked immunosorbent assay (ELISA) using Ready‐SET‐go kits (eBioscience, Inc., San Diego, CA). The detection levels of the kits (standard curve range) are as follows: IL‐10 Kit, 32–4000 pg/mL; TNF‐*α* Kit, 8–1000 pg/mL. The levels of cytokines in renal tissue were normalized by protein concentration (measured by Bio‐Rad detergent compatible protein assay method) (Cat. No. # 500‐0112; Bio‐Rad, Hercules, CA).

#### Immunohistochemical staining for IL‐10 and TNF‐α

Immunohistochemical staining of paraffinized sections (5 *μ*m) of kidney was performed for TNF‐*α* expression using anti‐mouse TNF‐*α* antibody (cat. no. # ab9739; Abcam, Cambridge, MA) and for IL‐10 expression using polyclonal goat anti‐mouse rIL‐10 antibody (cat. no. # AF519; R & D Systems Inc., Minneapolis, MN). Measurement of expression intensity of these cytokines in renal tissue was performed using NIS Elements software (Nikon Instruments Inc, New York) (Holladay et al. 2011; Yang et al. 2011).

#### Measurement of renal hemodynamic and excretory parameters

Urine volume (V) was measured gravimetrically. Blood and urine samples collected during systemic infusion of drugs were analyzed for measurements of inulin, PAH, and sodium/potassium concentrations as previously reported (Shahid et al. 2008, 2010) to determine GFR, RBF, urinary sodium excretion rate (U_Na_V), and fractional excretion of sodium (FE_Na_).

### Calculations and statistical analysis

Results are expressed as means ± SE. Differences between basal and treatment values in the same set of experiments were analyzed by a paired Student's *t*‐test. Comparison of the data among the different sets of experiments was made by one‐way analysis of variance, followed by the Student–Newman–Keuls post hoc test for multiple comparisons. Differences were considered significant at *P* < 0.05. In the case of analysis of cytokine values, the undetected concentration of plasma level was considered zero for statistical analysis.

## Results

### IL‐10 levels in vehicle‐treated control as well as in l‐NAME‐treated mice

**Figure 2.**
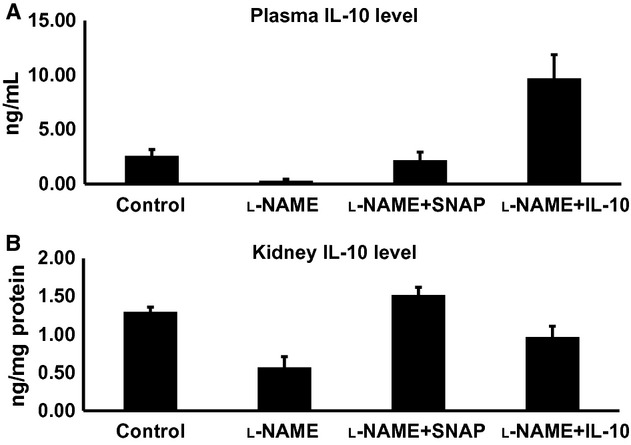
Levels of IL‐10 (interleukin‐10) in plasma (A) and in renal tissue (B) in response to l‐NAME (nitro‐l‐arginine methyl ester; 200 *μ*g/min/Kg) infusion with or without coinfusion of SNAP (S‐nitroso‐N‐acetylpenicillamine; a NO donor; 25 *μ*g/min/Kg) or exogenous IL‐10 (0.075 ng/min/g) in anesthetized mice. Values are means ± SE; *n* = 6 animals/group. *P <**0.05 versus control group; ^#^*P *<**0.05 versus l‐NAME group.

**Figure 3.**
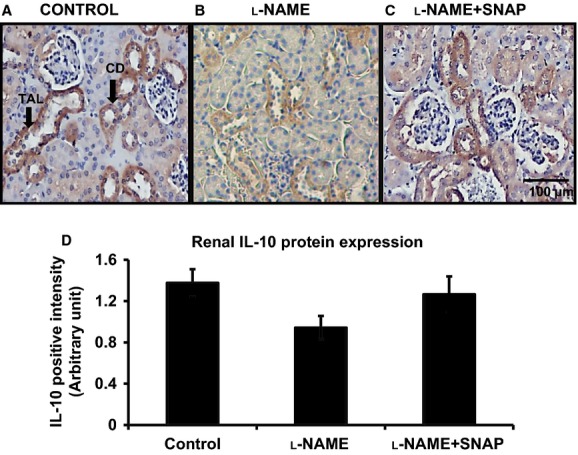
(A–C) Immunoexpression for interleukin‐10 (IL‐10) in renal tissues. IL‐10 protein is expressed in the renal tissue (mainly in the distal tubular cells) in control mice (A). Nitro‐l‐arginine methyl ester (l‐NAME) treatment reduces IL‐10 protein expression in the renal tissue (B). SNAP treatment in the presence of l‐NAME restored renal IL‐10 expression (C). TAL, Thick ascending limb of the loop of Henle; CD, collecting ducts. Microphotograph was taken using a 20× magnification objective. (D) illustrates the mean values of the comparative renal expression for IL‐10 protein in various conditions. Values are means ± SE; *n* = 6 animals/group. *P <**0.05 versus control group; ^#^*P *<**0.05 versus l‐NAME group.

Figure 2 depicts that IL‐10 is present in substantial concentration in plasma and renal tissue in control mice. IHC staining of renal sections from control mice also shows the presence of IL‐10 protein expression in distal nephron segments, mainly in the thick ascending limb of Henle's loop and collecting duct (Fig. 3A). Inhibition of NOS activity during l‐NAME infusion leads to reduction in IL‐10 in plasma as well as in renal tissue. There was also reduction in immunoexpression of IL‐10 protein in the renal sections (Fig. 3B and D). SNAP infusion in l‐NAME‐treated mice prevented the decrease in endogenous levels of IL‐10 in plasma as well as in renal tissue homogenates. The reduction in IL‐10 immunoexpression in renal section during l‐NAME infusion was also reversed by SNAP treatment (Fig. 3C and D). Exogenous infusion of mouse recombinant IL‐10 with l‐NAME reversed the plasma and renal levels of IL‐10 (Fig. 2A and B). However, it was noted that such infusion of IL‐10 (in group 4) resulted in plasma levels that were higher than the values in vehicle‐treated control groups. This is due to the infusion of a considerably high IL‐10 dose as it was intended to completely replenish the endogenous level of IL‐10 that was reduced during l‐NAME infusion. However, the modest effects in restoring the renal tissue level of IL‐10 may not be unexpected as this increased IL‐10 level in renal tissue is not from an endogenous source but derived from the plasma and may not be adequately reabsorbed in the tissue within this short period of infusion. Infusion of exogenous IL‐10 in l‐NAME‐treated mice did not alter the general localization (distal nephron segments) or the reduced pattern of IL‐10 protein expression as observed in mice with l‐NAME treatment alone.

### TNF‐α level after replacement of NO or IL‐10 during NOS‐inhibited condition

**Figure 4.**
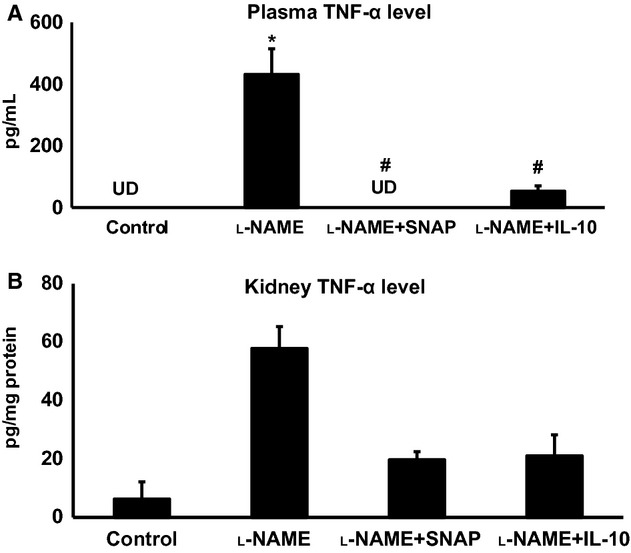
Levels of tumor necrosis factor (TNF‐*α*) in plasma (A) and in renal tissue (B) in response to nitro‐l‐arginine methyl ester (l‐NAME) infusion with or without coinfusion of SNAP (S‐nitroso‐N‐acetylpenicillamine; a NO donor) or IL‐10 in anesthetized mice. UD, undetected level. Values are means ± SE; *n* = 6 animals/group. *P <**0.05 versus control group (UD considered as zero value for statistical purpose); ^#^*P *<**0.05 versus l‐NAME group.

**Figure 5.**
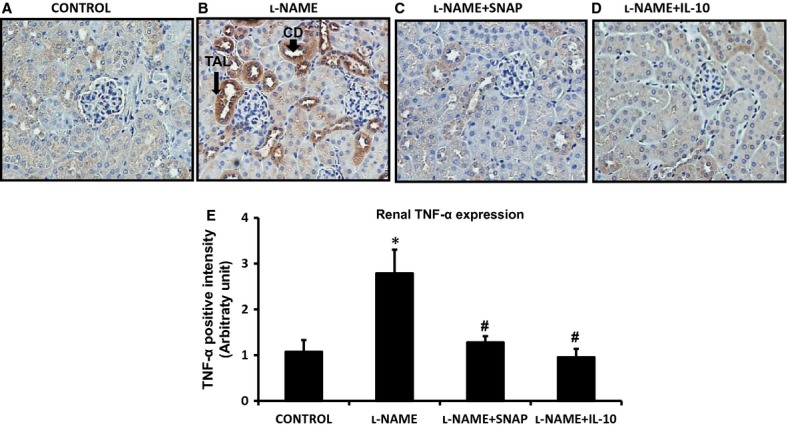
(A–D) Immunoexpression for TNF‐*α* in renal tissue. TNF‐*α* immunoexpression was minimal in the renal tissue slices of control mice (A), markedly present in the renal tissue (mainly in the loop of Henle and distal nephron segments) of l‐NAME‐treated mice (B), but not in mice coinfused with SNAP (C) and IL‐10 (D). TAL, Thick ascending limb of the loop of Henle; CD, collecting ducts. Microphotograph was taken using a 20× magnification objective. (E) illustrates mean values of the comparative renal expression of TNF‐*α* protein in various conditions. Values are means ± SE; *n* = 6 animals/group. *P <**0.05 versus control group, ^#^*P *<**0.05 versus l‐NAME group.

Plasma TNF‐*α* level was undetected in the control group (Fig. 4A). However, NOS inhibition in l‐NAME‐treated mice enhanced TNF‐*α* level both in plasma and in the renal tissues as shown in Figure 4. These results are similar to those reported earlier (Shahid et al. 2010). Interestingly, cotreatment of SNAP or IL‐10 with l‐NAME prevented this increase in TNF‐*α* level. IHC staining for TNF‐*α* in the renal section (Fig. 5A–D) indicates the presence of TNF‐*α*‐expressing cells in the kidney (mainly in the loop of Henle and distal nephron segments). TNF‐*α* protein expression was significantly higher in the renal section obtained from l‐NAME‐only‐treated mice (Fig. 5B and E). IL‐10 treatment prevented the l‐NAME‐induced increase in renal expression of TNF‐*α* (Fig. 5D and E). SNAP infusion also prevented the l‐NAME‐induced increase in renal TNF‐*α* immunoexpression (Fig. 5C and E). This suggests that restoration of IL‐10 by NO donor leads to inhibition of TNF‐*α* production during l‐NAME treatment. This result supports earlier reports by others suggesting that IL‐10 has a strong inhibitory effect on the production of TNF‐*α* (Florentino et al. 1991; Fouqueray et al. 1995).

### Effects of SNAP and IL‐10 treatment on the renal hemodynamic responses to l‐NAME infusion

NOS blockade by l‐NAME infusion resulted in marked increases in systemic arterial pressure among all the groups. As usual, this rise in arterial pressure by NO blockade was prevented by coinfusion of SNAP. However, this l‐NAME‐induced increase in arterial pressure remained unaltered during coinfusion of IL‐10 in these mice. Table 1 summarizes the systemic arterial pressure and renal hemodynamic responses to coinfusion of SNAP or IL‐10 with l‐NAME infusion. Only the averages of the values in the third (C3) and the fourth (C4) collection period are given in Table 1 to represent the group values obtained in these mice. As compared to the vehicle‐infused (group 1) mice, group 2 mice with only l‐NAME infusion had higher MAP and lower RBF without any significant difference in GFR. In the l‐NAME + SNAP‐treated mice, MAP was lower compared to l‐NAME‐alone‐treated mice, without significant differences in RBF and GFR. In the l‐NAME + IL‐10‐treated mice, no significant differences in MAP, RBF, or GFR were observed compared to l‐NAME‐alone‐treated mice.

**Table 1.** Renal hemodynamic and excretory responses to various drug infusions in anesthetized mice.

Parameters	Control (*n* = 6)	l‐NAME (*n* = 6)	l‐NAME + SNAP (*n* = 6)	l‐NAME + IL‐10 (*n* = 6)
Mean arterial pressure (mm Hg)	95 ± 5	118 ± 6*	94 ± 7*	121 ± 4
Renal blood flow (mL/min/g)	6.55 ± 0.32	4.79 ± 0.29*	5.06 ± 1.66	5.69 ± 0.41
Glomerular filtration rate (mL/min/g)	0.98 ± 0.06	0.87 ± 0.08	0.98 ± 0.2	1.15 ± 0.14
Urine flow (*μ*L/min/g)	8.22 ± 1.33	21.22 ± 2.60*	7.76 ± 1.61*	7.90 ± 1.06*
Urinary sodium excretion (*μ*mol/min/g)	0.99 ± 0.26	3.76 ± 0.66*	1.25 ± 0.33*	1.62 ± 0.26*
Fractional excretion of sodium (%)	0.68 ± 0.11	2.85 ± 0.38*	1.35 ± 0.54*	0.99 ± 0.19*

The values depicted here are the averages of the C3 and C4 collection periods in each group of mice as described in the experimental protocol. Values are means ± SE; *n* = 6 animals/group.

* *P* < 0.05 versus control group.

* *P* < 0.05 versus l‐NAME group.

### Effects of SNAP and IL‐10 treatment on the renal excretory responses to l‐NAME infusion

The absolute changes in the excretory function in response to l‐NAME infusion in the various groups of mice are given in Table 1. Only the averages of the values in the third (C3) and the fourth (C4) collection periods are given in Table 1 to represent the group values obtained in these mice. Compared to saline‐treated mice, l‐NAME–only‐treated mice had markedly higher urine flow, U_Na_V and FE_Na_. SNAP infusion in l‐NAME‐pretreated mice prevented these increases in urine flow, U_Na_V and FE_Na_ in response to l‐NAME infusion. Interestingly, IL‐10 infusion also prevented these l‐NAME‐induced increases in urine flow, U_Na_V and FE_Na_.

## Discussion

In this study, we have demonstrated that systemic NOS inhibition reduces the plasma and renal tissue levels of IL‐10 in mice which is associated with increases in TNF‐*α* level in respective tissues. Although TNF‐*α* level is undetected in plasma under control conditions, it is observed that IL‐10 is normally present in plasma and also IL‐10 protein is constitutively expressed in the renal tissue, mostly in the epithelial cells of distal nephron segments as demonstrated by IHC staining in this study. l‐NAME infusion strikingly reduces such protein expression in the renal tissue (Fig. 3), which is also associated with a marked increase in TNF‐*α* protein expression, mostly in the loop of Henle and other distal tubular segments (Fig. 5). Infusion of a NO donor compound (SNAP) during l‐NAME infusion prevented such changes in IL‐10 and TNF‐*α* level in plasma and in renal tissues indicating that NO deficiency is primarily responsible for the initiation of these alterations in the status of these inflammatory molecules. Although the renal tissue level of NO is not measured in the present study, previous studies conducted in our laboratory (Majid et al. 1998, 1999) had demonstrated that NOS inhibition by nitro‐l‐arginine (NLA) administration in anesthetized dogs decreases renal tissue NO concentration as measured by a NO measuring electrode inserted in the kidney. Infusion of SNAP in NLA‐pretreated dogs increased NO concentration in the renal tissue (Majid et al. 1998). Thus, it is conceivable that the changes in the renal tissue levels of inflammatory molecules (IL‐10 and TNF‐*α*) in response to l‐NAME and SNAP administration are related to changes in tissue NO levels. The findings in the present study demonstrate that a reduction in IL‐10 during NOS inhibition facilitates the enhancement of TNF production in the kidney. Infusion of exogenous IL‐10 to replenish its level that was minimized during l‐NAME infusion prevented the increase in the level of TNF‐*α* in plasma and renal tissues in response to NOS inhibition. In another preliminary study in our laboratory (Singh et al. 2013a), it was also noted that the levels of TNF‐*α* in the plasma and in the renal tissue are significantly higher in IL‐10 knockout (KO) mice than those in the corresponding wild‐type (WT) strain of mice. These findings support the notion that the production of TNF‐*α* is normally suppressed by endogenous IL‐10 production. Interestingly, it was also noted that there were increases in eNOS protein expression in the renal tissue as well as in urinary excretion of NO metabolites, nitrate/nitrite in the IL‐10 KO mice indicating that a change in NO production was not directly affecting the TNF‐*α* production. These data suggest that the reduction in IL‐10 level is the primary factor responsible for the enhancement in endogenous TNF‐*α* generation during NOS inhibition.

Contrasting results have been reported in the literature linking the regulation of IL‐10 production by NO. Earlier in an in vitro study (Qiu et al. 1999), it was shown that the lipopolysaccharide (LPS)‐induced enhancement in IL‐10 production in mouse alveolar macrophage (AM) cells was attenuated in a similar fashion when these cells were cotreated with either a NOS inhibitor, *S*‐methylisothiourea sulfate (SMT) or a NO donor compound, SNAP. However, a later study using the rat model of cerebral ischemia demonstrated that the enhanced circulating level of IL‐10 was lowered by chronic treatment with l‐NAME (Clarkson et al. 2007). On the other hand, it was also noted in a recent study in our laboratory (Singh et al. 2013a) that in IL‐10 gene KO mice, there were increases in eNOS protein expression in the renal tissue as well as in the urinary excretion of NO metabolites, nitrate/nitrite in the IL‐10 KO mice. However, the present study demonstrates clearly that a substantive level of IL‐10 is constitutively present in plasma as well as in renal tissues in mice which can be reduced with acute inhibition of NOS activity. These findings suggest that NO plays an important role in the regulation of endogenous production of IL‐10 that maintains the homeostasis with respect to the inflammatory status in the healthy individual. A reduction in endogenous IL‐10 production has been recently linked to the development of angiotensin II (ANG II)‐induced hypertension and inflammation in mice which can be partially attenuated by replacement of T‐regulatory (Treg) cells, a common source of IL‐10 production (Kassan et al. 2011; Matrougui et al. 2011). Hypertensive conditions induced by many factors such as chronic ANG II administration are usually associated with NO deficiency (Hermann et al. 2006) which could have resulted in diminution of Treg cells and thus, caused a generalized reduction in IL‐10 production in those conditions. Thus, it is possible that NO maintains the endogenous levels of IL‐10 by inducing the proliferation and sustained survival of Treg cells (Niedbala et al. 2006)**.** However, further experiments are required to elucidate the exact mechanistic link between productions of NO and IL‐10.

Many previous studies (Clarke et al. 1998; Rajasingh et al. 2006; Smallie et al. 2010) had implicated the anti‐inflammatory role of IL‐10 as a suppressor of TNF‐*α* production by monocyte/macrophage cells. These studies had addressed this mechanistic relationship between IL‐10 and TNF‐*α* production which suggested both transcriptional and posttranscriptional/translational mechanisms to mediate such anti‐inflammatory effect of IL‐10. IL‐10 inhibits nuclear factor kappa B activation (Clarke et al. 1998) and also inhibits transcription of TNF‐*α*‐related cytokine gene (Smallie et al. 2010). IL‐10 interferes with the activation potential of the p38/MAPK pathway, which is required to activate TNF‐*α* translation. Inhibition of p38/MAPK was shown to affect TNF*α*‐mRNA translation by inhibiting LPS‐induced polysome coupling of TNF*α*‐mRNA in macrophages (Rajasingh et al. 2006). A recent study in vitro as well in vivo (Rossato et al. 2012) also demonstrates a central role of miR‐187, an IL‐10‐dependent miRNA in the physiological regulation of IL‐10‐driven anti‐inflammatory responses. It showed that TNF‐*α* production in the macrophages is reduced when miR‐187 expression is enhanced and its production is increased significantly when miR‐187 expression is silenced. The results of this study in vivo also demonstrate further the immune downregulating action of IL‐10 as an anti‐inflammatory agent on the endogenous production of proinflammatory cytokine, TNF‐*α* (Florentino et al. 1991).

The effects of NOS inhibition/reduction in IL‐10 level may not be specific to TNF‐*α* level alone but also to other proinflammatory molecules as demonstrated in another study in our laboratory (Singh et al. 2012). It was observed that acute treatment with l‐NAME decreased IL‐10 level and increased IL‐6, IL‐1B, and monocyte chemoattractive protein‐1 levels in the renal tissue. Chronic l‐NAME treatment in rats (Miguel‐Carrasco et al. 2008) also caused increases in plasma levels of proinflammatory cytokines such as IL‐6, IL‐1*β,* and TNF‐*α*, as well as their RNA expression in the heart. It has been reported earlier that IL‐10 KO mice have increased IL‐6 mRNA expression in the aorta along with increased TNF‐*α* mRNA expression upon administration of ANG II (Didion et al. 2009). In two separate recent studies (Singh et al. 2013a,b, we have also shown that renal tissue levels of TNF‐*α* and IL‐6 are significantly higher in mice lacking the genes for eNOS (Singh et al. 2013b) and IL‐10 (Singh et al. 2013a) compared to corresponding WT mice.

Several studies (Florentino et al. 1991; Qiu et al. 1999) earlier have postulated that anti‐inflammatory cytokine, IL‐10 exhibits an inhibitory effect on the production of proinflammatory cytokines such as TNF‐*α*. As IL‐10 level is decreased in hypertensive conditions as mentioned earlier, this may be primarily involved in the increased production of TNF‐*α* in many models of experimental hypertension such as ANG II‐dependent hypertensive rats (Ferreri et al. 1997, 1998; Niedbala et al. 2006) and Dahl salt‐sensitive rats (Gu et al. 2006; Elmarakby et al. 2008). IL‐10 inhibits the production of TNF‐*α* through the inhibition of nuclear localization of NF‐kB (Wang et al. 1995). IL‐10 can also induce TNF‐*α* mRNA destabilization mediated via suppression of mitogen‐activated protein kinase activation and inhibition of expression of mRNA‐stabilizing protein HuR, family member of RNA‐binding proteins (Rajasingh et al. 2006).

The possibility that the increase in TNF‐*α* production during l‐NAME administration may be related secondarily to an increase in arterial pressure during NO inhibition is unlikely as it is observed in the present experiments that infusion of exogenous IL‐10 attenuates TNF‐*α* level without alteration in systemic arterial pressure (Table 1). It is also interesting to note that there seems to be a striking association between renal TNF‐*α* levels (Figs. 4, 5) and urine flow (Table) that may indicate an involvement of the fluid shear stress in enhanced TNF‐*α* production by tubular epithelial cells as shown in an in vitro study (Sedor et al. 1993). However, the contribution of such possible shear stress in the renal tubules on the production of TNF‐*α* has not yet been determined conclusively in any in vivo study earlier. In our recent studies (Singh et al. 2013a,b, we have observed that the renal tissue levels of TNF‐*α* and IL‐6 are significantly higher in KO mice lacking the genes for eNOS and IL‐10 compared to corresponding WT mice though the urine flow rate is similar in both KO and WT mice. As we have demonstrated earlier (Shahid et al. 2008), TNF‐*α* infusion induces diuretic and natriuretic responses in anesthetized mice which could be blocked in mice pretreated with TNF‐*α* receptor antagonist, etanercept. Moreover, l‐NAME‐induced diuretic and natriuretic responses were also prevented in mice pretreated with etanercept (Shahid et al. 2010). The findings clearly indicate that the diuresis and natriuresis in response to l‐LAME infusion is the downstream effect of TNF‐*α*, not a reverse phenomenon as may be suggested in that study using in vitro preparations (Miravète et al. 2012). However, an independent effect of flow on renal TNF‐*α* production needs to be examined in an in vivo setting more comprehensively.

In conclusion, the findings in the present study demonstrate that NO deficiency primarily leads to the suppression of endogenous IL‐10 production, thus, minimizing its immune downregulating action on the production of TNF‐*α*.

## Conflict of Interest

None declared.
